# MUC16 (CA125): tumor biomarker to cancer therapy, a work in progress

**DOI:** 10.1186/1476-4598-13-129

**Published:** 2014-05-29

**Authors:** Mildred Felder, Arvinder Kapur, Jesus Gonzalez-Bosquet, Sachi Horibata, Joseph Heintz, Ralph Albrecht, Lucas Fass, Justanjyot Kaur, Kevin Hu, Hadi Shojaei, Rebecca J Whelan, Manish S Patankar

**Affiliations:** 1 Department of Obstetrics and Gynecology, University of Wisconsin-Madison, Madison, WI 53792, USA; 2 Department of Obstetrics and Gynecology, University of Iowa, Iowa City, IA 52242, USA; 3 Department of Animal Sciences, University of Wisconsin-Madison, Madison, WI 53706, USA; 4 Department of Chemistry and Biochemistry, Oberlin College, Oberlin, OH 44074, USA

**Keywords:** Ovarian cancer, CA125, MUC16, Cancer biomarker, Metastasis, Immunesupression, Natural killer cells

## Abstract

Over three decades have passed since the first report on the expression of CA125 by ovarian tumors. Since that time our understanding of ovarian cancer biology has changed significantly to the point that these tumors are now classified based on molecular phenotype and not purely on histological attributes. However, CA125 continues to be, with the recent exception of HE4, the only clinically reliable diagnostic marker for ovarian cancer. Many large-scale clinical trials have been conducted or are underway to determine potential use of serum CA125 levels as a screening modality or to distinguish between benign and malignant pelvic masses. CA125 is a peptide epitope of a 3–5 million Da mucin, MUC16. Here we provide an in-depth review of the literature to highlight the importance of CA125 as a prognostic and diagnostic marker for ovarian cancer. We focus on the increasing body of literature describing the biological role of MUC16 in the progression and metastasis of ovarian tumors. Finally, we consider previous and on-going efforts to develop therapeutic approaches to eradicate ovarian tumors by targeting MUC16. Even though CA125 is a crucial marker for ovarian cancer, the exact structural definition of this antigen continues to be elusive. The importance of MUC16/CA125 in the diagnosis, progression and therapy of ovarian cancer warrants the need for in-depth research on the biochemistry and biology of this mucin. A renewed focus on MUC16 is likely to culminate in novel and more efficient strategies for the detection and treatment of ovarian cancer.

## Introduction

CA125 is best known as a biomarker to monitor epithelial ovarian cancer and for the differential diagnosis of pelvic masses [1,2]. Serum levels of CA125 are routinely monitored in patients with ovarian cancer, and an increase from an individualized nadir concentration is a prognostic indicator of cancer recurrence. An extensive body of work has been published on the interpretation of CA125 assay results, with particular focus given to CA125 in ovarian cancer patients undergoing therapy [1-12]. Understanding of the clinical applicability of the CA125 assay has come from basic, translational, and clinical studies conducted since the early 1980’s [13-20]. However, what remains underappreciated is that the assay to measure CA125 in clinical samples is limited by an incomplete understanding of the biochemical structure of this antigen. Here, we will review the current knowledge of the structure of CA125 and provide critical appraisal of factors that may interfere with accurate and sensitive measurement of this biomarker.

CA125 is a repeating peptide epitope of the mucin MUC16 [21,22], which promotes cancer cell proliferation and inhibits anti-cancer immune responses [23-26]. Our discussion of the biological role of MUC16 will focus on its importance in cancer cell signaling, metastasis, regulation of immune responses and on anti-cancer therapeutic strategies that target this mucin. A major goal of this review is to highlight gaps in our knowledge of the biochemistry and biology of MUC16/CA125 to provide a framework of unanswered questions to be addressed in future studies.

## CA125 as a clinical biomarker

CA125 has been extensively investigated as a biomarker in three separate clinical scenarios: (1) as a screening test for the early detection of ovarian cancer, (2) to distinguish between benign and malignant disease in pre- and post-menopausal women presenting with pelvic masses, and (3) to monitor response to therapy in women with ovarian cancer.

### CA125 as a screening modality for ovarian cancer

An increase (beyond a cut-off of 30–35 U/ml) is generally observed in blood samples that are serially obtained from women with ovarian cancer [27]. Therefore, for screening, longitudinal monitoring of serum CA125 levels is likely to be more useful than a single measurement. The Risk of Ovarian Cancer Algorithm (ROCA) predicts the probability of ovarian cancer based on such longitudinal monitoring of CA125 [27,28]. ROCA allows triaging of patients into groups with low, intermediate and high risk of ovarian cancer.

ROCA was developed after retrospective analysis of serum samples obtained from an ovarian cancer screening trial of 5550 women conducted in Sweden [29,30]. This trial concluded that serum CA125 monitoring will be most beneficial in post-menopausal women. In this cohort, the serum CA125 test provides sensitivity and specificity of 91 and 94.5%, respectively.

The largest fully reported ovarian cancer screening trial that used CA125 as a biomarker (in combination with ultrasonography) was conducted in the United Kingdom with a cohort of 22,000 women [31]. A total of 41 women tested positive for ovarian cancer based on CA125 and ultrasound. Only eleven of these 41 cases were found to be truly positive for ovarian cancer after subsequent surgical investigation. Eight women who had screened negative for ovarian cancer developed the disease 12–22 months after the initial screen.

Enthusiasm for the success of combination of CA125 assay and transvaginal ultrasound screening is tempered by the negative results from the Prostate, Lung, Colorectal, and Ovarian Cancer (PLCO) trial [32-34]. In this randomized control trial of 78,216 subjects, women in the intervention arm were screened for CA125 levels and transvaginal ultrasound. There was no improvement in mortality (the primary endpoint of this study) in this trial. The PLCO trial did not employ ROCA. A retrospective analysis [35] has suggested that the use of ROCA in PLCO would not have led to statistically significant mortality benefit of screening, in part because of the long delay (one year) between screens and the absence of a standardized diagnostic algorithm [32].

Given the low prevalence of ovarian cancer in the general population, it is estimated that a good clinical trial to develop a screening paradigm for ovarian cancer would require recruitment of >150,000 women [30,36]. The United Kingdom Collaborative Trial of Ovarian Cancer Screening (UKCTOCS) accrued a total of 202,638 post-menopausal women between 2001 and 2005 [37]. Subjects were randomized into (1) control, (2) serum CA125 plus transvaginal ultrasound, and (3) transvaginal ultrasound only groups [38]. The ROCA was used to triage women in the second group to low, intermediate and high-risk categories. Subjects in the intermediate-risk category were monitored for CA125 levels every 4 months, and if they continued to show increased risk for ovarian cancer, were monitored by ultrasound scans. The subjects in the high-risk category underwent transvaginal ultrasound scans [38]. Final results from this trial are expected in 2015. Interim analysis from this trial has led to the promising finding that 47.1% of women testing positive after combined assessment with CA125 test and transvaginal ultrasound had stage I or II disease [38]. The combined use of ROCA and ultrasound, strict guidelines for follow up in 3–4 months after elevated CA125 is detected and a well-coordinated plan for screening patients across all participating sites are advantages of the UKCTOCS over the PLCO study design that are expected to contribute to successful identification of a screening strategy for ovarian cancer [35,39]. Survival data from the UKCTOCS trial is expected in 2015.

### CA125 as a diagnostic marker for ovarian cancer

The majority of patients with ovarian cancer are periodically assayed for CA125. An increase in concentration of CA125 above a nadir is an indicator of disease recurrence [40-42]. Another important potential application of the CA125 assay is in distinguishing benign pelvic masses from ovarian cancer [43]. The Risk of Malignancy Index (RMI) combines serum CA125 concentrations with ultrasound and menopausal status to distinguish between benign disease and cancer [44]. Recent studies suggest that a Risk of Ovarian Malignancy Algorithm (ROMA) incorporating CA125 and HE4 levels in serum is likely to produce a test of high sensitivity and specificity in identifying ovarian cancer patients [45-47].

Currently, HE4 is the only biomarker, other than CA125, that has been approved by the U.S. Food and Drug Administration as a diagnostic marker for ovarian cancer [48,49]. Another algorithm, OVA1, uses CA125, apolipoprotein A1, transthyretin, transferrin and β2-microglobulin and takes into account data from ultrasound imaging and menopausal status of patients [50].

Mesothelin, osteopontin, CA15.3, CA19.9, AFP, KLK6 and several others have been investigated and suggested as potential biomarkers for ovarian malignancy. A recent multi-center effort to test the sensitivity and specificity of panels of markers conducted using the serum samples obtained from PLCO has been reported [51]. None of the panels of markers tested in this trial improve on the sensitivity and specificity of CA125 in screening or ovarian cancer diagnosis [52,53]. The conclusion from the important trials and studies described in this section is that CA125 continues to be, and for the foreseeable future will persist as, the predominant and most clinically useful biomarker for ovarian cancer. It is therefore imperative that significant effort be put forth to clearly understand the chemical nature of this antigen with the express intent of improving the widely used CA125 assay.

## Biochemistry of CA125

CA125 was first identified in a screen of monoclonal antibodies raised against the ovarian cancer cell line OVCA433 [13,54]. One antibody, OC125, recognized an antigen in ovarian cancer specimens which was designated as CA125 [13-17,55]. Several antibodies, M11, VK8 and OV197 among them, were subsequently developed against CA125 [56,57]. Based on their binding specificities, CA125-specific antibodies are grouped as belonging to the OC125, M11 or OV197 families [58-61].

Initial gel filtration experiments suggested that CA125 had a molecular weight as high as 2 million Da [62]. Hanisch and colleagues demonstrated that CA125 activity was contained in a high molecular weight mucinous fraction isolated from human milk [63]. Isopycnic density gradient centrifugation led to isolation of CA125 activity in a fraction with the buoyant density of 1.41 g/ml, a density that is typically observed for most human mucinous glycoproteins [63].

A more definitive study on the molecular characterization of CA125 by Davis et al. showed that CA125 isolated from OVCA433 cells had a buoyant density of 1.42 g/ml whereas the antigen in human serum and milk had a density of 1.46 g/ml and 1.39 g/ml, respectively [64]. Davis and co-workers also performed size exclusion chromatography of OVCA433 cell culture supernatant on a Sepharose CL-4B column, a matrix with a size exclusion limit of 6 × 10^4^ – 2 × 10^7^ Da for globular proteins. These chromatography studies demonstrated that CA125 activity was contained in fractions having molecular masses of 200,000-1 million Da [64].

Importantly, the report by Davis et al. conclusively refuted a previous claim that CA125 was a carbohydrate epitope [63,64]. Oxidation of the CA125-containing fractions at pH 4.5 with 1 mM and 10 mM sodium periodate—conditions that oxidize vicinal hydroxyls of terminal sialic acid or those present in the entire oligosaccharide chains, respectively [65,66]—did not result in significant loss of OC125 binding to the antigen. Instead, exposure to heat and low pH (100°C, 3.3 pH), protease digestion, reduction and alkylation in buffer containing 4 M guanidine hydrochloride abrogated binding of OC125 to the antigen. These crucial observations suggested that CA125 was a conformationally dependent peptide epitope present in a high molecular weight mucin. Although some incremental advances in our understanding of the molecule were gained from important fundamental research conducted independently by Lloyd and O’Brien [20,56,57,62,67-70], major advance in our understanding of the biochemistry of CA125 was gained from the identification of MUC16 as the mucin that contained this antigen.

### MUC16, a large molecular weight membrane-spanning mucin

**Figure 1:**
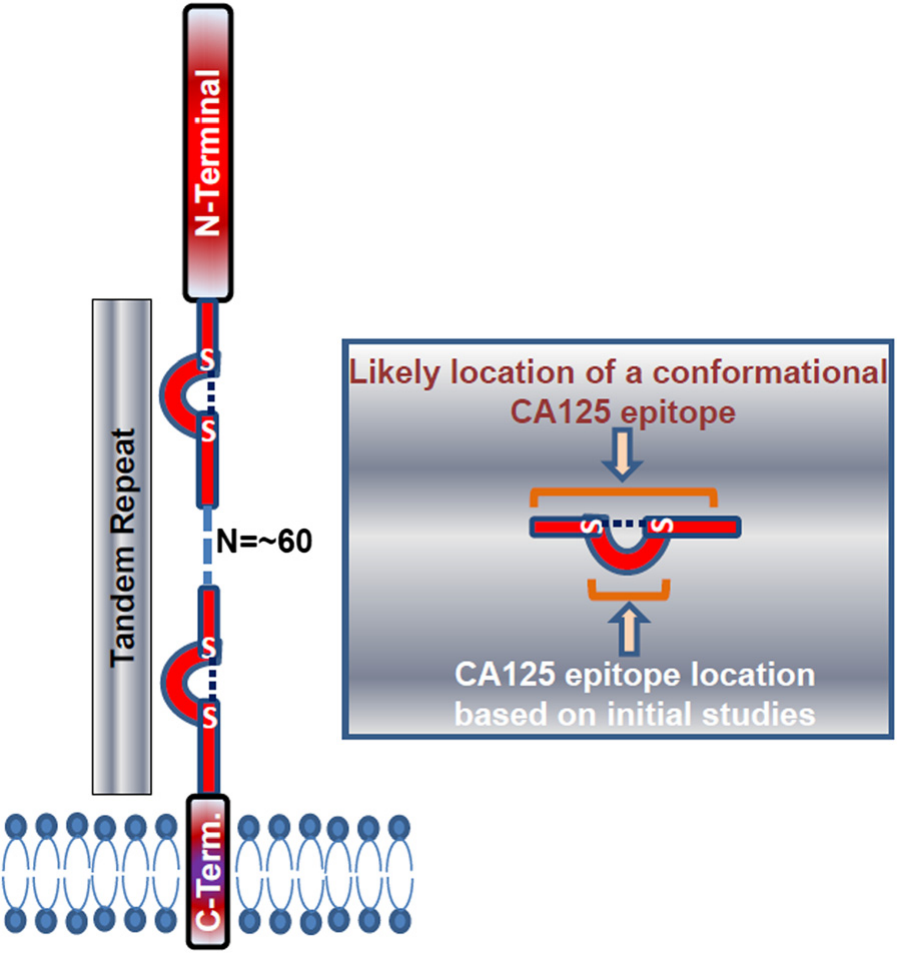
**MUC16 structure.** Model shows the three domains of MUC16 and potential location of the CA125 epitope in a tandem repeat.

Molecular cloning of MUC16 revealed it to be a mucinous glycoprotein with an average molecular weight between 3–5 million Da [21,22,71,72]. Similar to other membrane-spanning mucins, MUC16 (Figure 1) is composed of a tandem repeat region sandwiched between N-terminal and C-terminal domains [21,22]. The C-terminal domain is the smallest part of the molecule and is composed of 284 amino acids (Figure 1). While it has been proposed that this C-terminal domain can be phosphorylated under specific conditions, conclusive proof is lacking [21,73,74].

The N-terminal region is composed of 12,068 amino acids [71]. Other than potential sites for both N-linked and O-linked glycosylation, there are no major structural features identified in this domain. The tandem repeat domain is composed of up to 60 repeats (Figure 1). Each repeat has 156 amino acids [21]. The primary amino acid sequence in each repeat is not identical but is homologous. Two conserved cysteines, at positions 59 and 79 of each repeat, are proposed to have structural significance [21]. These cysteines could form intra- as well as inter-molecular disulfide bonds. While the intramolecular disulfide bonds could form loops within each MUC16 molecule, intermolecular disulfide linkages may contribute to formation of an extracellular matrix [71].

Evidence supporting the location of CA125 antigen in the MUC16 tandem repeat came from two initial studies where the tandem repeats were expressed in *E. coli* or in human ovarian cancer cell lines [21,72]. In the first study, the 11^th^ MUC16 tandem repeat (R11) was expressed and isolated from *E. coli *[21]. This recombinant R11 protein was recognized by the three anti-CA125 antibodies M11, OC125 and OV197. In another study, a recombinant protein containing three of the MUC16 tandem repeats was produced in two cell lines—SW626 and SKOV-3—that do not express MUC16 [72]. The recombinant proteins expressed in these cell lines were detected by M11 and OC125 but not by the VK8 antibody. This finding was interesting because VK8 was initially classified as an M11-type antibody, but studies with the recombinant MUC16 fragments demonstrated clear differences in the epitope specificities of M11 and VK8 [72].

Digestion of the recombinant R11 tandem repeat by the endoproteases Lys-C or Asp-D completely destroyed the CA125 epitope as demonstrated by the observation that the resulting fragments were not detected by the OC125 or M11 antibodies [21]. It was primarily this one experiment that led to the prevalent viewpoint that the CA125 epitope is located in the 21-amino acid loop of the tandem repeats formed by disulfide bridging of cysteines located at positions 59 and 79. Recent experiments conducted by us and by Bressan et al. [75] have led us to believe that this model is inaccurate and that the CA125 epitope has not been sufficiently characterized.

In our experiments we did not observe binding of OC125 and M11 antibodies to a synthetic 21-mer peptide sequence (Peptide 1) comprising the loop region shared by eight of the 60 MUC16 tandem repeats. We also investigated OC125 and M11 binding to three variants of Peptide 1 that differ in single amino acids (C21A, Peptide 3; P8S, Peptide 4) or in two amino acids (P8S and C21A, Peptide 5). These variants were selected because their sequences are also found in the MUC16 repeats (Ser appears in position 8 in ~25% of tandem repeats) or they produce specific modifications in the secondary structures of the peptides (replacing Cys with Ala removes the possibility of intramolecular disulfide bonding) [76]. In five independent assay protocols—Silicon Photonic Microring Resonator Immunoassay, Surface Plasmon Resonance Immunoassay, ELISA, Competitive ELISA and Affinity Probe Capillary Electrophoresis—none of these four peptides were recognized by OC125 and M11 antibodies.

Not all of the MUC16 repeats are recognized to the same extent by these antibodies [75]. Recombinant proteins containing either R2, R7, R9, R11, R25, or R51 repeats were recognized by M11 in Western blot assays. However, only a subset of these repeats (R9, R11, R25, and R51) were detected strongly by OC125 and a partially overlapping subset (R2, R9, R25, and R51) were detected by OV197 antibodies. Deletion mutants of the 156 amino acid R25 repeat that are missing residues 129–156 from the C-terminal end retain binding by OC125, M11 and OV-197. However, deletion of the amino acids 1–30 from the N-terminal end of this truncated mutant abrogated binding by all three antibodies. Thus the CA125 epitope is likely localized between amino acids 1–128 of the MUC16 repeats [75]. However, any further refined characterization of the CA125 epitope has not been achieved.

Incidentally, the region 1–128 of R25 contains the Sea urchin Enterokinase and Agrin (SEA) domain [71,77,78]. In fact BLAST protein homology search shows the presence of SEA domains in each of the MUC16 tandem repeats (Additional file 1). In addition, one SEA domain is also located in the C-terminal region of the mucin. While MUC1 and some other mucins are known to contain a single or limited number of SEA domains, such extensive presentation of these structural units is unique to MUC16 among all of the identified mammalian mucins. One SEA domain from the murine Muc16 ortholog has been structurally characterized [78]. While SEA domains can exhibit autoproteolytic activity, any potential importance of this domain in the shedding of MUC16 is not clear.

Antibodies that recognize continuous epitopes are likely to bind sequences that are composed of not more than 15–22 amino acids of which 5–6 residues are critical as they provide maximum binding energy [79]. However, deletion of even 30 amino acids from the N- or the C-terminal end of the 128 amino acid recombinant repeat domain abrogates antibody binding [75]. These results have prompted the suggestion that the CA125 epitope is not continuous but is instead a discontinuous epitope that is dependent on the secondary conformational structure of the MUC16 tandem repeat [75]. While this suggestion is consistent with our studies with the C-loop peptides, its acceptance relies on the proposal that detection of the 1–128 deletion mutant by the CA125 antibodies in western blots follows renaturation during transfer to the nitrocellulose membrane [75]. One important conclusion drawn from our studies with the peptides and those reported by Bressan et al. is that the CA125 epitope most likely does not reside in the loop region of the tandem repeat as predicted initially [21,75]. The implications of this conclusion are serious as it indicates that even though the CA125 assay is routinely used to monitor the vast majority of patients with serous ovarian cancer, the exact molecular nature of the antigen is not accurately characterized.

The CA125 II assay currently used to measure serum concentrations of this biomarker in patients uses M11 as a capture antibody and OC125 as a tracer [13,56,70]. Studies with recombinant R11 indicate that binding of OC125 to this antigen is enhanced several-fold after pre-binding of M11 [61]. Whether enhancement—or inhibition—of OC125 binding is also observed with other recombinant repeats of MUC16, or the entire molecule in patient sera, needs to be determined. A corollary to this statement is that since OC125 binding is sensitive to the molecular environment of its epitope, other factors such as post-translational modification may influence detection of CA125 in patient sera.

**Figure 2:**
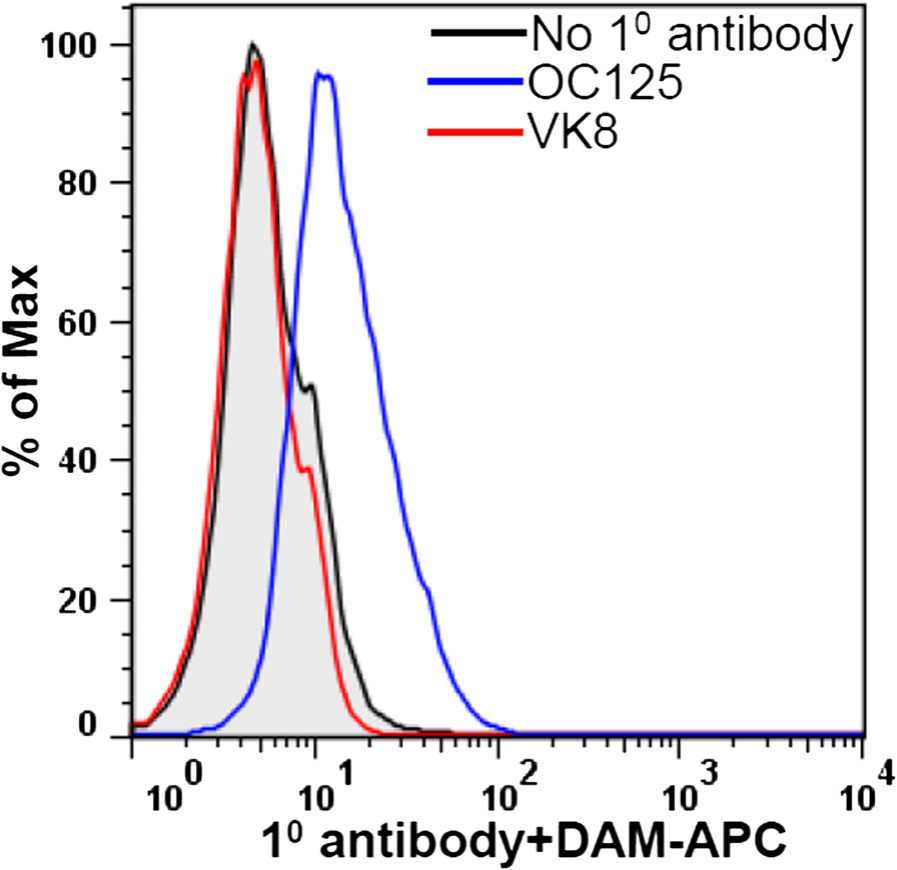
**OC125 weakly binds to cells that are generally considered negative for MUC16.** SKOV-3 cells (identity confirmed by STR analysis) were stained with the primary (1°) antibodies OC125 or VK8 followed by incubation with allophycocyanine (APC)-labeled donkey anti-mouse (DAM) secondary antibody. Binding of the antibodies to SKOV-3 cells was determined on a LSR-II flow cytometer. RT-PCR using MUC16 primers previously reported [178], was used to determine expression of MUC16 in OVCAR-3, ECC-1 and SKOV-3 cells (data not shown).

**Figure 3:**
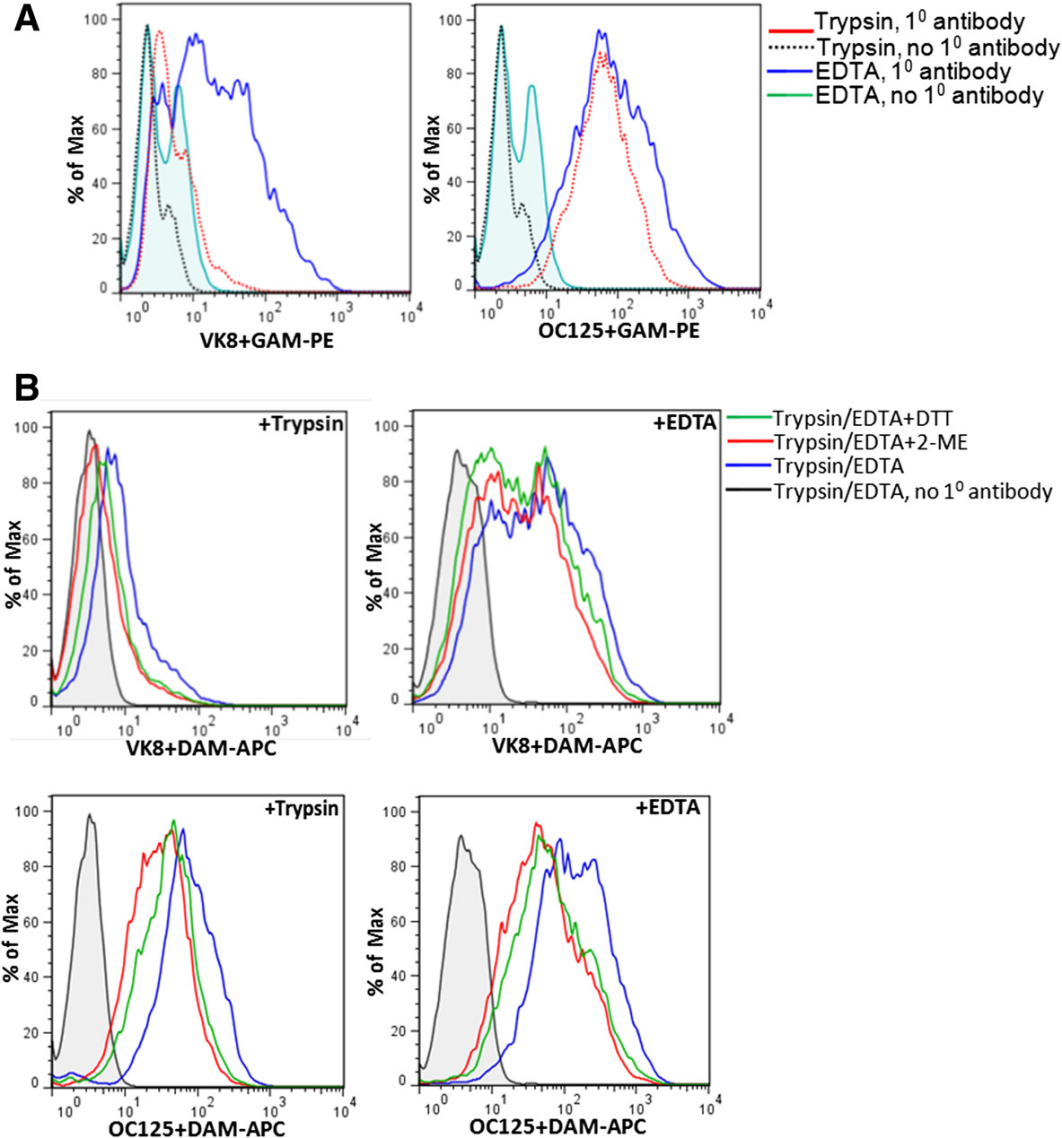
**Cell surface MUC16 is sensitive to proteolysis. A**, Binding of OC125 and VK8 to OVCAR-3 cells that were harvested with trypsin or EDTA containing media was determined by flow cytometry. A significant decrease in VK8 binding to cells harvested after trypsin treatment was observed whereas under these conditions, OC125 binding was less affected. **B**, Reduction of the trypsinized OVCAR-3 cells with dithiothreitol (DTT) or 2-mercaptoethanol (2-ME) did not result in additional loss of OC125 binding to the cells. In all experiments, a fluorescently tagged goat anti-mouse antibody was used for detection.

In our experiments with ovarian cancer cell lines, we have observed that OC125 weakly binds to the surface of cells—SKOV-3 and A2780—that are generally considered to be non-expressors of MUC16. However, under the same conditions another anti-CA125 antibody, VK8, which was used in the initial cloning experiments [22,57], does not recognize SKOV-3 (Figure 2). The marginal binding of OC125 to SKOV-3 cells corresponds to weak expression of MUC16 as determined by RT-PCR (data not shown).We observe that trypsin digestion significantly reduces VK8 binding to MUC16-expressing cells, but only slightly reduces OC125 binding to cells under the same conditions (Figure 3). We tested the possibility that even after trypsin digestion the OC125 epitopes remain bound to the cell surface via disulfide bridges. Mild treatment of the cells with mercaptoethanol or dithiothreitol to release disulfide-bound moieties did not decrease OC125 binding to the trypsinized OVCAR-3 cells (Figure 3).

If CA125 is indeed a discontinuous epitope, it is not clear if the O- or N-glycosylation of the tandem repeat will influence binding of OC125, M11 or OV197. Most studies characterizing the MUC16 repeats have been conducted with recombinant proteins produced in bacterial cells [21,75] and therefore lacking native glycosylation. Similarly our 21mer peptides, assembled by solid-phase peptide synthesis, are not glycosylated. After initial characterization of the glycosylation of MUC16 [80], there has not been a robust and systematic characterization of the glycan chains attached to each domain of the mucin or of the glycan site occupancy in mucin molecules produced by tumor versus normal epithelial cells. Attempts to identify disease-specific MUC16 glycoforms by glycomic analysis are producing interesting results that may enhance the diagnostic potential of this mucin [81-88]. However, with the enormous potential diversity in glycan chains and glycoforms a significant effort will be required to fully understand the post-translational status of this mucin and its effect on detection in the CA125 assay and systemic half-life of the MUC16 glycoforms.

Mucins in systemic circulation are rapidly processed by the reticulo-endothelial cells, leaving behind only a fraction of the mucins in circulation to be detected in clinical assays such as the CA125 test [89]. The combined effects of MUC16 clearance—reducing the amount of circulating MUC16 to miniscule levels—and the inability of the antibodies to uniformly bind all tandem repeats and isoforms—undercounting circulating MUC16—suggests that the CA125 assay significantly underestimates the concentration of this biomarker. New generation of reagents and assay protocols that can capture and more accurately quantify a larger cohort of the MUC16 molecules will likely add to the clinical significance of this biomarker.

To date, the majority of the anti-MUC16 antibodies raised against full-length mucin recognize regions within the tandem repeat domains [58,59]. An explanation for the bias toward antibodies that recognize the tandem repeat region of MUC16 is not available but is likely due to higher immunogenicity of these epitopes. Antibodies against defined epitopes that are outside of the tandem repeat region (against peptides from the carboxy terminal end [90,91], for example) will serve an important purpose in modifying the CA125 assays and also in attempts to understand the biology of this mucin.

## Biological role of MUC16

**Figure 4:**
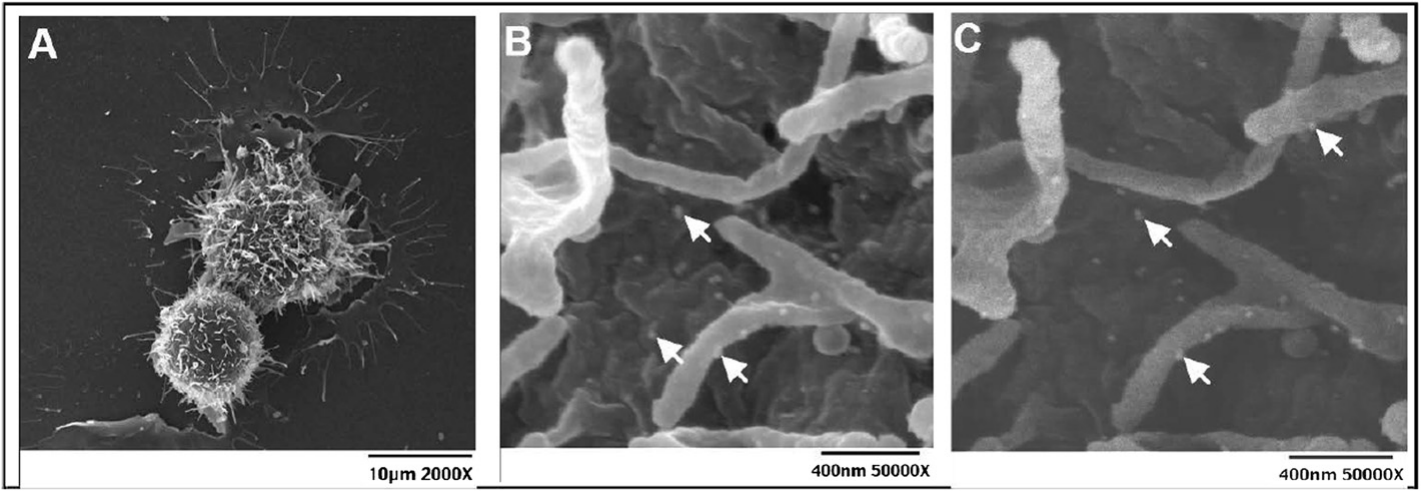
**MUC16 on the surface of ovarian cancer cells.** OVCAR-3 cells were labeled with VK8 followed by colloidal gold nanoparticles conjugated with goat anti-mouse secondary antibody. **A**, low magnification secondary electron image of two labeled OVCAR-3 cells. **B**, Scanning Electron Microscopy (SEM) image of OVCAR-3 showing colloidal gold nanoparticles binding to cell surface and microvilli. **C**, Back scattered electron image of same cell surface shown in **B**, clearly showing the colloidal gold nanoparticles. Bright spots (some indicated by bright arrows) in **B** and **C** are the colloidal gold nanoparticles. OVCAR-3 cells are not labeled with colloidal gold nanoparticles in the absence of VK-8 (data not shown).

MUC16 is expressed by normal bronchial, endometrial, ovarian and corneal epithelial cells and has evolved from the proteoglycan, agrin [24,92-98]. A transmembrane region anchors the mucin in the cell membrane and as a result the mucin can be detected on the cell surface (Figure 4).

MUC16 is released from the cell surface following proteolytic cleavage at a site presumably 50 amino acids upstream of its transmembrane segment [21]. As a result the proteolytically released ectodomain of MUC16 is not significantly different in molecular weight than the cell surface-bound intact mucin.

Neutrophil elastase, MMP-7, MMP-9 and bacterial metalloprotease (ZmpC) can release the ectodomain of MUC16 [99-101]. Post-translational modifications (especially glycosylation) around the site of cleavage are thought to regulate the release of the ectodomains of these mucins [100]. Post-translational modifications around the cleavage site may be a variable that impacts the sensitivity of the CA125 assay.

In corneal epithelial cells, MUC16-Galectin-3 interaction has been shown to serve as a barrier for bacterial and viral infections in ocular epithelia [102,103]. These are the first studies to show a potential biological role for MUC16 in non-cancer cells. However, knockdown of murine Muc16 does not result in any obvious functional deficit [104,105]. Since these knockout mice were maintained in a pathogen free environment and were not otherwise challenged with chemical or biological agents, the potential impact of Muc16 knockdown in specific pathological states remains unknown. Further exploration into the normal physiologic role(s) of human and murine MUC16 is warranted.

### Immunoprotection of cancer cell cells

Innate immune cells, Natural Killer (NK) cells and monocytes are unable to attack tumor cells expressing high levels of MUC16 [106]. Knockdown of MUC16 results in increased lysis of the ovarian cancer cells by the cytolytic NK cells. The mechanism by which MUC16 inhibits NK cell-mediated killing of cancer cells is under investigation. One proposal is that MUC16 acts as a barrier that prevents interaction between the NK cells and their targets [106]. NK cells require physical contact with the target cells for cytolysis to occur. NK cell immunologic synapse is defined by polarization of cytoskeletal and cell signaling molecules along with cytolytic granules of the effector cells to the site of contact [107-109]. The NK cell cytolytic granules are released into the target cells, triggering cell death via apoptosis. MUC16 with its ~5 million Da molecular size is expected to have a linear length of 1–5 μm [106,110]. Due to its extended structure and overall negative charge (due to the presence of terminal sialic acid residues [80]), MUC16 may inhibit intimate interactions between NK and cancer cells. This situation is analogous to the mechanism of immuneprotection demonstrated for MUC4 [111].

**Figure 5:**
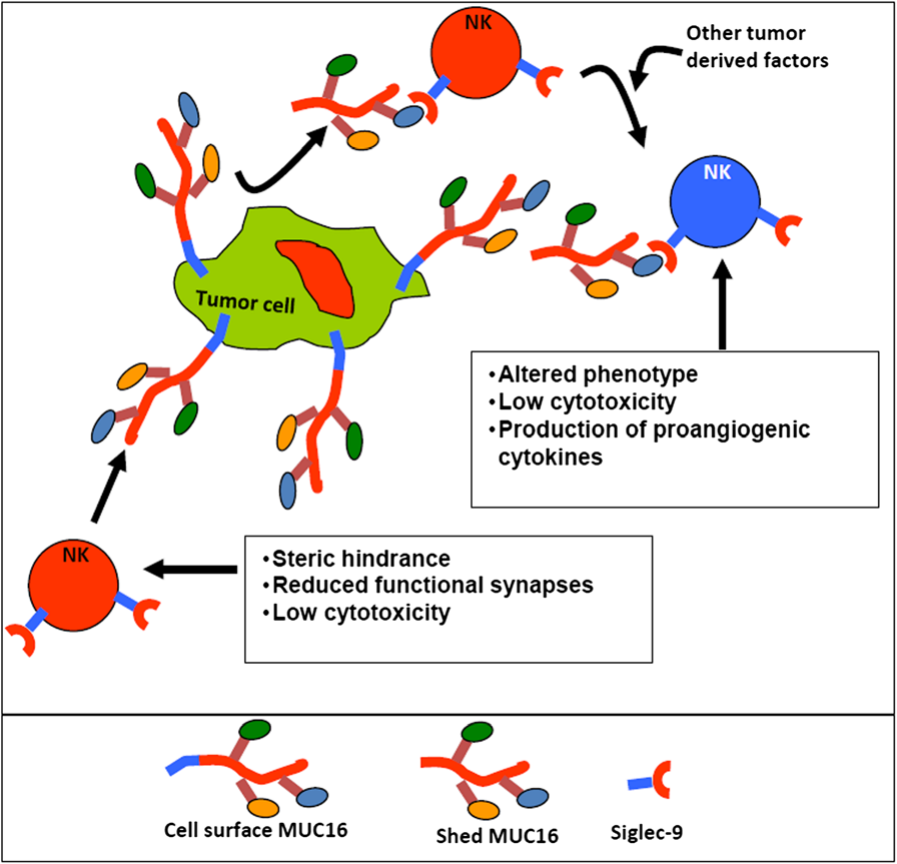
**Model for MUC16-induced NK cell inhibition.** MUC16 released from tumors binds to naïve NK cells (shown in red) and along with other tumor derived factors induces a phenotypic and functional change. The altered NK cells (shown in blue) secrete cytokines that promote angiogenesis. The cell surface bound MUC16, on the other hand, acts as an anti-adhesive mucin and blocks the interaction between the NK cells and ovarian tumor cells thereby preventing cancer cell cytolysis.

Imunoprotective effect by MUC16 may also arise from its interaction with the NK cell inhibitory receptor, Siglec-9 (Figure 5) [112]. NK cells express both activating (NKG2D, CD16, DNAM-1, for example) and inhibitory receptors (Killer Immunoglobulin-like Receptors (KIR’s), Siglec-9, and Siglec-7, for example) on their cell surface. Interaction of activating and inhibiting receptors with their corresponding ligands on target cells leads to either activation or inhibition of the NK cell cytolytic response (reviewed in [113] and other articles). The interplay between the activating and inhibitory receptors controls the cytolytic activity of NK cells.

Siglecs are a class of inhibitory receptors that bind to negatively charged sialic acid ligands [114-119]. MUC16 oligosaccharides carry sialic acid in a terminal α2-3 linkage [80]. Oligosaccharides terminated with α2-3-linked sialic acids are recognized as ligands by the inhibitory Siglec-9 receptor [120]. Our work demonstrates that MUC16-Siglec-9 interaction protects cancer cells from NK cell attack. Siglec-9 is also expressed on monocytes and MUC16 promotes binding of monocytes to cancer cells. Upon binding to its ligand, Siglec-9 is phosphorylated on its Immunoreceptor Tyrosine-based Inhibition Motif (ITIM) tail, triggering an inhibitory signaling cascade that results in inhibition of the NK cell response [121]. Ovarian cancer cells are therefore likely to be protected from NK cell and monocyte attack due to the negative signaling induced via MUC16-Siglec-9 interaction (Figure 5). This type of protection can occur from interaction of the NK and monocyte Siglec-9 with MUC16 on the surface of cancer cells and also from circulating MUC16 molecules cleaved from the cancer cells via proteolysis.

MUC16 protects ovarian cancer cells from naïve unstimulated NK cells as well as IL-2 stimulated NK cells. A recent study has implicated MUC16 in inhibiting target cell killing via Antibody-dependent Cell-Mediated Cytotoxicity (ADCC) [122], suggesting that the mucin may dampen the response of immunotherapeutic antibodies.

NK cells preferentially target ovarian tumor cells that express low levels of MUC16 on their cell surface [106]. As a result, tumor cells surviving NK cell attack are high expressors of MUC16 that are more resistant to immune attack. We have proposed that NK cells contribute to immune editing [123-125] by selectively lysing cancer cells expressing lower levels of MUC16. As a result, those cancer cells with high levels of MUC16, the subset that is most likely to resist immune attack, survive.

### Pro-metastasis role of MUC16

Immune editing in favor of cancer cells expressing higher levels of MUC16 may also increase peritoneal metastasis of ovarian tumors [126-129]. MUC16 binds to mesothelin, a GPI-anchored glycoprotein found on the surface of mesothelial cells and also overexpressed by ovarian tumors, with an apparent Kd of 5 nM [23,130,131]. MUC16-mesothelin interaction allows tumor cells to bind to themselves (likely increasing tumor mass at metastatic sites) and also allows attachment of ovarian cancer cells to the mesothelial lining [130]. Removal of N-glycans from MUC16 attenuates its interaction with mesothelin [132]. Mesothelin, similar to other members of its superfamily, contains superhelical ARM-type repeats that could potentially recognize carbohydrate ligands presented by MUC16 [132].

MUC16, via its sialofucosylated oligosaccharides, binds to both E- and L-selectin even under shear stress conditions [133]. Contribution of MUC16-selectin interaction to ovarian cancer progression or metastasis is not known. Even after MUC16 knockdown, E- and L-selectin binding is observed to cancer cells, presumably via other glycolipid, mucin, or glycoprotein ligands [133]. Therefore, interpretation of results MUC16-selectin interactions and their possible roles in metastasis are complicated by presence of such redundant binding mechanisms.

### MUC16 associated cell signaling

MUC1 and MUC4 undergo auto-cleavage during their synthesis to generate α- and β-subunits [134-136]. The β-subunit of MUC1 and MUC4 is involved in intracellular signaling. For example, EGF stimulation causes the MUC1 β-subunit to signal via PI3K and MAPK [137,138] MUC1 β-subunit also interacts with p53, NF-κB, β-catenin and STAT1 [139,140].

Phosphorylation of the cytoplasmic portion of C-terminal domain of MUC16 increases shedding of the ectodomain of this mucin [74]. Specific serine, threonine and tyrosine residues in the cytoplasmic tail of MUC16 have been proposed as likely candidates for phosphorylation [21].

Knockdown of MUC16 inhibits in vitro and in vivo proliferation of cancer cells [141-143] MUC16-triggered activation of STAT3 via JAK2 results in increasing cancer cell proliferation. On the other hand, knockdown of MUC16 causes cell cycle arrest (either in G2/M or G1 phase) and apoptosis [143,144].

Expression of the C-terminal domain of the mucin in the MUC16^neg^ SKOV-3 cells increases their proliferation with 2–3 fold increase in tumor weight and a significant decrease in survival of tumor-bearing mice [142]. MUC16 knockdown causes a decrease in the expression of matrix metalloproteases (MMP-2), a class of proteolytic enzymes that plays a crucial role in cancer cell metastasis [144-146]. A recent report suggests that the cytoplasmic tail of MUC16 interacts with Src-family kinases and induces E-cadherin-mediated cell invasion and migration [147].

### MUC16 is overexpressed but not associated with decreased survival or chemoresistance

**Figure 6:**
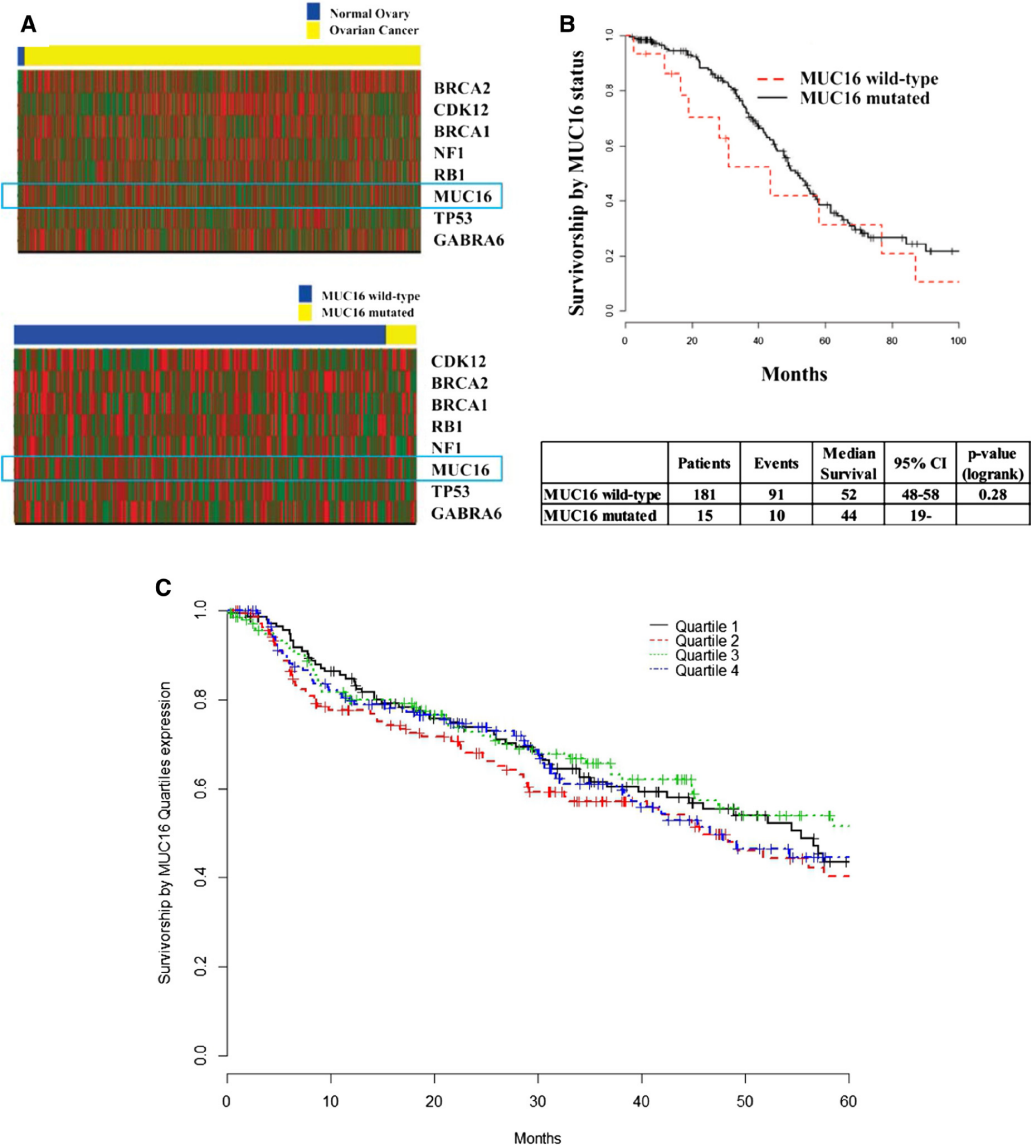
**Analysis of data on MUC16 available through TCGA analysis of ovarian cancer samples. A** TCGA data set [148] was analyzed to determine differential expression of prominent cancer-related genes between normal ovarian tissue and ovarian carcinoma (heat map in top panel). Although MUC16 is upregulated in the cancer samples (expression in cancer specimens is double than in normal) this difference is not significant (p-value = 0.1), probably because there were only 8 normal samples in TCGA dataset. MUC16 is compared to other prominent mutated genes reported in the original TCGA report [148]. Of these genes, only BRCA2 is differentially expressed between normal and cancer (p-value <0.001) samples. Analysis of mutated and wild-type MUC16 expression in samples from ovarian cancer patients listed in TCGA is shown in the heat map in the lower panel. Mutated MUC16 is also mildly over-expressed as compared to wild-type mucin (expression Wild-type/Mutated = 0.94), however this difference is not significant (p-value = 0.84). MUC16 is compared to other genes reported to be highly mutated at the original report of TCGA. None of the other genes is differentially expressed between MUC16-wild-type and MUC16-mutated ovarian cancer specimens, indicating no association between MUC16 mutation status and key genes expression in this dataset (p-value <0.05). **B**, Survival of ovarian cancer patients with wild-type and mutated MUC16 was compared. Although a trend was observed suggesting worse outcome in patients with mutated MUC16, the difference was not statistically significant (p-value = 0.28). **C**, MUC16 (both mutated and wild-type) expression was divided into quartiles. Survival of patients in each of these quartiles was not significantly different.

Silencing of MUC16 increased the sensitivity of OVCAR-3 cells to cisplatin and doxorubicin but not to paclitaxel [141]. However, bioinformatics analysis of MUC16 expression and mutations available through The Cancer Genome Atlas (TCGA) [148] does not support a major effect of MUC16 on overall survival in ovarian cancer patients (Figure 6). No correlation is observed between MUC16 expression and resistance to chemotherapy in the ovarian cancer cohorts. Of the patients on whom MUC16 data has been deposited in TCGA, mutations in the mucin were identified in fifteen of 196 patients (Additional file 2). Patients with mutated MUC16 had a slightly lower survival than patients with wild-type MUC16; however the difference was not statistically significant (Figure 6).

### MUC16 as a target for therapy

The lack of significant effect of MUC16 observed in our analysis of TCGA data is inconsistent with the effect of MUC16 observed in in vitro and in vivo studies with ovarian and breast cancer cells, described above, where the expression of this mucin was attenuated. This dichotomy may potentially arise from the fact that only eight healthy donor samples are reported in the TCGA database for ovarian cancer. We estimate that data from 40 individuals each in the healthy control and ovarian cancer group will be required to attain 90% power to demonstrate the effect of differential MUC16 gene expression on patient survival. If no correlation between MUC16 expression and survival is demonstrated in a larger study, an intriguing possibility that will have to be considered is that MUC16 may be a factor whose presence (in terms of biological significance) is felt only by its absence (as shown by the knockdown experiments). If true, this model suggests that significant effect on ovarian tumors may be achieved by the application of small molecule agents and other strategies for in vivo knockdown of MUC16 or inhibition of its biological function. In this scenario, tumors will be subject to increased apoptosis, decreased proliferation, increased sensitivity to chemotherapy, and reduced efficiency in subverting innate immune responses and immunotherapeutic agents. In this context it is interesting to note that microRNA-200c regulates expression of both MUC4 and MUC16 [149].

### MUC16 as a target for immunotherapy

Anti-mesothelin antibodies that interfere with mesothelin-MUC16 binding have been developed as immunotherapeutic agents [150-153]. A novel immunoadhesin, HN125, contains the MUC16 binding epitope of mesothelin fused to the Fc portion of human IgG1 antibody has been developed [154,155]. However, additional work is required to increase the potency of HN125 to produce a stronger anti-cancer response.

Oregovomab and Abgovomab are two antibodies developed for ovarian cancer immunotherapy. Oregovomab, (B43.13, OVAREX) is a mouse anti-MUC16 antibody developed for the in vivo detection of ovarian tumors [156]. A retrospective analysis of results trials designed to test the use of B43.13 as an imaging agent suggested increased survival in patients developing a human anti-mouse antibody (HAMA) response [157]. Repeated administration of the anti-CA125 antibodies results in increased anti-tumor T cell responses and the generation of anti-idiotypic antibodies [158-165]. These positive results led to the testing of Oregovomab in larger clinical trials in ovarian cancer patients [166,167]. Overall the results of these trials were disappointing as no benefit of Oregovomab was observed.

Abgovomab has met a similar fate in clinical trials. Abgovomab is an anti-idiotype antibody that was developed as a targeting agent against MUC16 [168-172]. Here again, clinical trials showed no difference in the overall survival of ovarian cancer patients as compared to patients in the control arm of the study [173-176].

One potential reason for Oregovomab and Abgovomab being unsuccessful in inhibiting tumor growth is that these antibodies may conjugate with the circulating ectodomain of MUC16 reducing the amount of antibody available to target the cancer cells. Additionally, shedding of the ectodomain may release the antibodies bound to the cell surface associated mucin. The rate at which the ectodomain is shed and the factors controlling the proteolysis of MUC16 are not clearly defined. It will be interesting to determine the efficacy of treatment regimens that combine anti-MUC16 antibodies with agents that can inhibit MUC16 release in controlling the growth of ovarian tumors.

### Targeting MUC16 with antibody-drug conjugates

Two anti-MUC16 antibodies, 3A5 and 11D10, were developed and conjugated to the cytotoxic drug Monomethyl auristatin E (MMAE). 3A5 targets MUC16 tandem repeats and is more effective than 11D10 in delivering MMAE to cancer cells and inducing cell death. The 3A5-MMAE conjugate (now referred to as DMUC5754A) is being tested in a Phase I clinical trial. Twenty two of the 44 patients recruited in this trial received DMUC5754A [177]. The antibody drug conjugate produced minimal toxicity and, importantly, one patient showed complete response and five patients experienced a reduction in tumor. These results are encouraging and support further development of MUC16-targeted therapies for cancer.

## Conclusion

Recent studies on MUC16 indicate that this mucin is not only important because it contains the biomarker CA125 but also for its role in contributing to ovarian tumor growth and metastasis. The complex biochemical structure of this mucin continues to provide major challenges to efforts being undertaken to make improvements to the CA125 assay and to understand the biological role of MUC16. The complexity of this antigen however also provides multiple opportunities that can be exploited to develop a test that can monitor this mucin even at low concentrations and, as the encouraging results with DMUC5754A suggest, developing novel anti-cancer therapeutic strategies. A sustained and systematic effort will be required to fully understand and exploit MUC16 to realize a benefit for patients with ovarian and other malignancies.

## Abbreviations

CA125: Cancer Antigen 125; MMAE: Monomethyl Auristatin E; MUC16: Mucin 16; NK: Natural killer cells; PLCO: Prostate, lung, colorectal and ovarian cancer; RMI: Risk of malignancy index; ROCA: Risk of ovarian cancer algorithm; ROMA: Risk of ovarian malignancy algorithm; SEA: Sea urchin Enterokinase and Agrin; TCGA: The cancer genome atlas; UKCTOCS: United Kingdom Collaborative Trial of Ovarian Cancer Screening.

## Competing interests

The authors declare that they have no competing interests.

## Authors’ contributions

MF conducted the flow cytometry experiments; AK conducted flow cytometry experiments and MUC16 mutation analysis; JGB conducted the TCGA data analysis and helped with writing of the manuscript; SH, JH and RA helped with obtaining the electron microscopy data; LF and JK conducted MUC16 mutation analysis; KH helped with the analysis of antibody binding to CA125 peptides; HS assisted in the writing of the manuscript; RJW and MSP conceptualized the idea for this review and wrote the manuscript. All authors read and approved the final manuscript.

## Supplementary Material

Additional file 1 BLAST analysis showing location of SEA domains in each of the repeat and the C-terminal domain of MUC16. Click here for file

Additional file 2 MUC16 mutations listed in TCGA data on ovarain cancer samples. Click here for file
