# Supplementary material for: MUC16 (CA125): tumor biomarker to cancer therapy, a work in progress
Source: Mol Cancer. 2014 May 29;13:129. doi: 10.1186/1476-4598-13-129 (PMC4046138; doi:10.1186/1476-4598-13-129)
Supplement: Additional file 1 — BLAST analysis showing location of SEA domains in each of the repeat and the C-terminal domain of MUC16. [file 1476-4598-13-129-S1.docx]

**Additional file 1**. BLAST analysis showing location of SEA domains in each of the repeat and the C-terminal domain of MUC16. Every tandem repeat and the C-terminal domain is identified as containing the same SEA domain, pfam01390, which is a member of the superfamily cl02507. This SEA domain is a good match for amino acids 4-114 of each MUC16 tandem repeat, as indicated by the high bit scores and E-values approaching 0. It is to be noted that the SEA domain (ID: pfam01390) does not contain the two cysteine residues that are conserved in all MUC16 tandem repeats and are thought to form the C-loops via disulfide bonding. Another important distinction is the absence in the SEA of a four amino acid sequence that is found in the MUC16 tandem repeat C-loop (shown from BLAST analysis at the end of this Supplementary table .) The C-terminal domain of MUC16 also contains one SEA domain. This C-terminal domain also contains a C-loop region similar to those found in the tandem repeats. The C-loop of the C-terminal domain also contains four amino acids that are not found in the SEA primary sequence.

| **Repeat #/Domain** | **Amino Acids** | **ID as SEA** | **Conserved Domain length** | **bitscore** | **E-value** | **Start** | **End** |
| --- | --- | --- | --- | --- | --- | --- | --- |
|  |  |  |  |  |  |  |  |
| R1 | 1638-1793 | [pfam01390](http://www.ncbi.nlm.nih.gov/entrez/query.fcgi?cmd=Search&doptcmdl=GenPept&db=cdd&term=pfam01390) | 107 | 88.2 | 6.33E-23 | 4 | 114 |
| R2 | 1794-1950 | [pfam01390](http://www.ncbi.nlm.nih.gov/entrez/query.fcgi?cmd=Search&doptcmdl=GenPept&db=cdd&term=pfam01390) | 107 | 84.74 | 1.49E-21 | 4 | 114 |
| R3 | 1953-2108 | [pfam01390](http://www.ncbi.nlm.nih.gov/entrez/query.fcgi?cmd=Search&doptcmdl=GenPept&db=cdd&term=pfam01390) | 107 | 90.52 | 7.85E-24 | 4 | 114 |
| R4 | 2109-2263 | [pfam01390](http://www.ncbi.nlm.nih.gov/entrez/query.fcgi?cmd=Search&doptcmdl=GenPept&db=cdd&term=pfam01390) | 107 | 86.28 | 3.47E-22 | 4 | 114 |
| R5 | 2264-2419 | [pfam01390](http://www.ncbi.nlm.nih.gov/entrez/query.fcgi?cmd=Search&doptcmdl=GenPept&db=cdd&term=pfam01390) | 107 | 87.05 | 1.64E-22 | 4 | 114 |
| R6 | 2420-2575 | [pfam01390](http://www.ncbi.nlm.nih.gov/entrez/query.fcgi?cmd=Search&doptcmdl=GenPept&db=cdd&term=pfam01390) | 107 | 89.75 | 1.79E-23 | 4 | 114 |
| R7 | 2576-2731 | [pfam01390](http://www.ncbi.nlm.nih.gov/entrez/query.fcgi?cmd=Search&doptcmdl=GenPept&db=cdd&term=pfam01390) | 107 | 83.2 | 4.83E-21 | 4 | 114 |
| R8 | 2732-2887 | [pfam01390](http://www.ncbi.nlm.nih.gov/entrez/query.fcgi?cmd=Search&doptcmdl=GenPept&db=cdd&term=pfam01390) | 107 | 85.89 | 4.01E-22 | 4 | 114 |
| R9 | 2888-3043 | [pfam01390](http://www.ncbi.nlm.nih.gov/entrez/query.fcgi?cmd=Search&doptcmdl=GenPept&db=cdd&term=pfam01390) | 107 | 86.66 | 2.57E-22 | 4 | 114 |
| R10 | 3044-3199 | [pfam01390](http://www.ncbi.nlm.nih.gov/entrez/query.fcgi?cmd=Search&doptcmdl=GenPept&db=cdd&term=pfam01390) | 107 | 85.89 | 4.79E-22 | 4 | 114 |
| R11 | 3200-3355 | [pfam01390](http://www.ncbi.nlm.nih.gov/entrez/query.fcgi?cmd=Search&doptcmdl=GenPept&db=cdd&term=pfam01390) | 107 | 92.44 | 1.50E-24 | 4 | 114 |
| R12 | 3356-3511 | [pfam01390](http://www.ncbi.nlm.nih.gov/entrez/query.fcgi?cmd=Search&doptcmdl=GenPept&db=cdd&term=pfam01390) | 107 | 85.12 | 7.42E-22 | 4 | 114 |
| R13 | 3512-3667 | [pfam01390](http://www.ncbi.nlm.nih.gov/entrez/query.fcgi?cmd=Search&doptcmdl=GenPept&db=cdd&term=pfam01390) | 107 | 83.97 | 2.27E-21 | 4 | 114 |
| R14 | 3668-3823 | [pfam01390](http://www.ncbi.nlm.nih.gov/entrez/query.fcgi?cmd=Search&doptcmdl=GenPept&db=cdd&term=pfam01390) | 107 | 85.89 | 4.01E-22 | 4 | 114 |
| R15 | 3824-3979 | [pfam01390](http://www.ncbi.nlm.nih.gov/entrez/query.fcgi?cmd=Search&doptcmdl=GenPept&db=cdd&term=pfam01390) | 107 | 92.06 | 2.33E-24 | 4 | 114 |
| R16 | 3980-4135 | [pfam01390](http://www.ncbi.nlm.nih.gov/entrez/query.fcgi?cmd=Search&doptcmdl=GenPept&db=cdd&term=pfam01390) | 107 | 78.57 | 2.33E-19 | 4 | 114 |
| R17 | 4136-4293 | [pfam01390](http://www.ncbi.nlm.nih.gov/entrez/query.fcgi?cmd=Search&doptcmdl=GenPept&db=cdd&term=pfam01390) | 107 | 86.66 | 2.19E-22 | 4 | 114 |
| R18 | 4295-4450 | [pfam01390](http://www.ncbi.nlm.nih.gov/entrez/query.fcgi?cmd=Search&doptcmdl=GenPept&db=cdd&term=pfam01390) | 107 | 80.12 | 7.44E-20 | 4 | 114 |
| R19 | 4451-4606 | [pfam01390](http://www.ncbi.nlm.nih.gov/entrez/query.fcgi?cmd=Search&doptcmdl=GenPept&db=cdd&term=pfam01390) | 107 | 92.83 | 1.11E-24 | 4 | 114 |
| R20 | 4607-4762 | [pfam01390](http://www.ncbi.nlm.nih.gov/entrez/query.fcgi?cmd=Search&doptcmdl=GenPept&db=cdd&term=pfam01390) | 107 | 90.13 | 9.45E-24 | 4 | 114 |
| R21 | 4763-4918 | [pfam01390](http://www.ncbi.nlm.nih.gov/entrez/query.fcgi?cmd=Search&doptcmdl=GenPept&db=cdd&term=pfam01390) | 107 | 93.6 | 4.55E-25 | 4 | 114 |
| R22 | 4919-5074 | [pfam01390](http://www.ncbi.nlm.nih.gov/entrez/query.fcgi?cmd=Search&doptcmdl=GenPept&db=cdd&term=pfam01390) | 107 | 91.67 | 2.84E-24 | 4 | 114 |
| R23 | 5075-5230 | [pfam01390](http://www.ncbi.nlm.nih.gov/entrez/query.fcgi?cmd=Search&doptcmdl=GenPept&db=cdd&term=pfam01390) | 107 | 87.05 | 1.67E-22 | 4 | 114 |
| R24 | 5231-5386 | [pfam01390](http://www.ncbi.nlm.nih.gov/entrez/query.fcgi?cmd=Search&doptcmdl=GenPept&db=cdd&term=pfam01390) | 107 | 81.66 | 1.98E-20 | 4 | 114 |
| R25 | 5336-5491 | [pfam01390](http://www.ncbi.nlm.nih.gov/entrez/query.fcgi?cmd=Search&doptcmdl=GenPept&db=cdd&term=pfam01390) | 107 | 85.12 | 8.60E-22 | 4 | 114 |
| R26 | 5492-5646 | [pfam01390](http://www.ncbi.nlm.nih.gov/entrez/query.fcgi?cmd=Search&doptcmdl=GenPept&db=cdd&term=pfam01390) | 107 | 68.17 | 1.88E-15 | 4 | 114 |
| R27 | 5647-5802 | [pfam01390](http://www.ncbi.nlm.nih.gov/entrez/query.fcgi?cmd=Search&doptcmdl=GenPept&db=cdd&term=pfam01390) | 107 | 85.12 | 9.86E-22 | 10 | 114 |
| R28 | 5803-5958 | [pfam01390](http://www.ncbi.nlm.nih.gov/entrez/query.fcgi?cmd=Search&doptcmdl=GenPept&db=cdd&term=pfam01390) | 107 | 82.43 | 9.62E-21 | 4 | 114 |
| R29 | 5959-6114 | [pfam01390](http://www.ncbi.nlm.nih.gov/entrez/query.fcgi?cmd=Search&doptcmdl=GenPept&db=cdd&term=pfam01390) | 107 | 81.66 | 2.03E-20 | 4 | 114 |
| R30 | 6115-6270 | [pfam01390](http://www.ncbi.nlm.nih.gov/entrez/query.fcgi?cmd=Search&doptcmdl=GenPept&db=cdd&term=pfam01390) | 107 | 89.75 | 1.61E-23 | 4 | 114 |
| R31 | 6271-6426 | [pfam01390](http://www.ncbi.nlm.nih.gov/entrez/query.fcgi?cmd=Search&doptcmdl=GenPept&db=cdd&term=pfam01390) | 107 | 80.5 | 5.02E-20 | 4 | 114 |
| R32 | 6427-6582 | [pfam01390](http://www.ncbi.nlm.nih.gov/entrez/query.fcgi?cmd=Search&doptcmdl=GenPept&db=cdd&term=pfam01390) | 107 | 89.36 | 2.43E-23 | 4 | 114 |
| R33 | 6583-6738 | [pfam01390](http://www.ncbi.nlm.nih.gov/entrez/query.fcgi?cmd=Search&doptcmdl=GenPept&db=cdd&term=pfam01390) | 107 | 92.83 | 1.12E-24 | 4 | 114 |
| R34 | 6739-6894 | [pfam01390](http://www.ncbi.nlm.nih.gov/entrez/query.fcgi?cmd=Search&doptcmdl=GenPept&db=cdd&term=pfam01390) | 107 | 92.83 | 1.07E-24 | 4 | 114 |
| R35 | 6895-7050 | [pfam01390](http://www.ncbi.nlm.nih.gov/entrez/query.fcgi?cmd=Search&doptcmdl=GenPept&db=cdd&term=pfam01390) | 107 | 78.19 | 4.13E-19 | 4 | 114 |
| R36 | 7051-7206 | [pfam01390](http://www.ncbi.nlm.nih.gov/entrez/query.fcgi?cmd=Search&doptcmdl=GenPept&db=cdd&term=pfam01390) | 107 | 71.64 | 9.36E-17 | 4 | 114 |
| R37 | 7207-7362 | [pfam01390](http://www.ncbi.nlm.nih.gov/entrez/query.fcgi?cmd=Search&doptcmdl=GenPept&db=cdd&term=pfam01390) | 107 | 87.43 | 1.24E-22 | 4 | 114 |
| R38 | 7363-7518 | [pfam01390](http://www.ncbi.nlm.nih.gov/entrez/query.fcgi?cmd=Search&doptcmdl=GenPept&db=cdd&term=pfam01390) | 107 | 67.4 | 3.22E-15 | 4 | 114 |
| R39 | 7519-7674 | [pfam01390](http://www.ncbi.nlm.nih.gov/entrez/query.fcgi?cmd=Search&doptcmdl=GenPept&db=cdd&term=pfam01390) | 107 | 77.42 | 7.22E-19 | 4 | 114 |
| R40 | 7675-7830 | [pfam01390](http://www.ncbi.nlm.nih.gov/entrez/query.fcgi?cmd=Search&doptcmdl=GenPept&db=cdd&term=pfam01390) | 107 | 67.79 | 2.35E-15 | 4 | 114 |
| R41 | 7831-7986 | [pfam01390](http://www.ncbi.nlm.nih.gov/entrez/query.fcgi?cmd=Search&doptcmdl=GenPept&db=cdd&term=pfam01390) | 107 | 84.35 | 1.83E-21 | 4 | 114 |
| R42 | 7987-8142 | [pfam01390](http://www.ncbi.nlm.nih.gov/entrez/query.fcgi?cmd=Search&doptcmdl=GenPept&db=cdd&term=pfam01390) | 107 | 68.56 | 1.46E-15 | 4 | 114 |
| R43 | 8143-8298 | [pfam01390](http://www.ncbi.nlm.nih.gov/entrez/query.fcgi?cmd=Search&doptcmdl=GenPept&db=cdd&term=pfam01390) | 107 | 86.28 | 3.18E-22 | 4 | 114 |
| R44 | 8299-8454 | [pfam01390](http://www.ncbi.nlm.nih.gov/entrez/query.fcgi?cmd=Search&doptcmdl=GenPept&db=cdd&term=pfam01390) | 107 | 68.56 | 1.46E-15 | 4 | 114 |
| R45 | 8455-8610 | [pfam01390](http://www.ncbi.nlm.nih.gov/entrez/query.fcgi?cmd=Search&doptcmdl=GenPept&db=cdd&term=pfam01390) | 107 | 87.05 | 1.50E-22 | 4 | 114 |
| R46 | 8611-8766 | [pfam01390](http://www.ncbi.nlm.nih.gov/entrez/query.fcgi?cmd=Search&doptcmdl=GenPept&db=cdd&term=pfam01390) | 107 | 67.02 | 4.10E-15 | 4 | 114 |
| R47 | 8767-8922 | [pfam01390](http://www.ncbi.nlm.nih.gov/entrez/query.fcgi?cmd=Search&doptcmdl=GenPept&db=cdd&term=pfam01390) | 107 | 86.66 | 2.66E-22 | 4 | 114 |
| R48 | 8923-9078 | [pfam01390](http://www.ncbi.nlm.nih.gov/entrez/query.fcgi?cmd=Search&doptcmdl=GenPept&db=cdd&term=pfam01390) | 107 | 66.25 | 8.01E-15 | 4 | 114 |
| R49 | 9079-9234 | [pfam01390](http://www.ncbi.nlm.nih.gov/entrez/query.fcgi?cmd=Search&doptcmdl=GenPept&db=cdd&term=pfam01390) | 107 | 85.12 | 7.59E-22 | 4 | 114 |
| R50 | 9235-9389 | [pfam01390](http://www.ncbi.nlm.nih.gov/entrez/query.fcgi?cmd=Search&doptcmdl=GenPept&db=cdd&term=pfam01390) | 107 | 68.17 | 1.91E-15 | 4 | 114 |
| R51 | 9390-9545 | [pfam01390](http://www.ncbi.nlm.nih.gov/entrez/query.fcgi?cmd=Search&doptcmdl=GenPept&db=cdd&term=pfam01390) | 107 | 86.28 | 3.59E-22 | 4 | 114 |
| R52 | 9546-9701 | [pfam01390](http://www.ncbi.nlm.nih.gov/entrez/query.fcgi?cmd=Search&doptcmdl=GenPept&db=cdd&term=pfam01390) | 107 | 66.25 | 1.00E-14 | 4 | 114 |
| R53 | 9702-9857 | [pfam01390](http://www.ncbi.nlm.nih.gov/entrez/query.fcgi?cmd=Search&doptcmdl=GenPept&db=cdd&term=pfam01390) | 107 | 86.28 | 2.80E-22 | 4 | 114 |
| R54 | 9858-10013 | [pfam01390](http://www.ncbi.nlm.nih.gov/entrez/query.fcgi?cmd=Search&doptcmdl=GenPept&db=cdd&term=pfam01390) | 107 | 89.75 | 1.55E-23 | 4 | 114 |
| R55 | 10014-10169 | [pfam01390](http://www.ncbi.nlm.nih.gov/entrez/query.fcgi?cmd=Search&doptcmdl=GenPept&db=cdd&term=pfam01390) | 107 | 86.66 | 2.25E-22 | 4 | 114 |
| R56 | 10170-10325 | [pfam01390](http://www.ncbi.nlm.nih.gov/entrez/query.fcgi?cmd=Search&doptcmdl=GenPept&db=cdd&term=pfam01390) | 107 | 88.98 | 2.81E-23 | 4 | 114 |
| R57 | 10326-10481 | [pfam01390](http://www.ncbi.nlm.nih.gov/entrez/query.fcgi?cmd=Search&doptcmdl=GenPept&db=cdd&term=pfam01390) | 107 | 83.97 | 2.56E-21 | 4 | 114 |
| R58 | 10532-10687 | [pfam01390](http://www.ncbi.nlm.nih.gov/entrez/query.fcgi?cmd=Search&doptcmdl=GenPept&db=cdd&term=pfam01390) | 107 | 90.9 | 5.11E-24 | 4 | 114 |
| R59 | 10688-10843 | [pfam01390](http://www.ncbi.nlm.nih.gov/entrez/query.fcgi?cmd=Search&doptcmdl=GenPept&db=cdd&term=pfam01390) | 107 | 91.67 | 2.73E-24 | 4 | 114 |
| R60 | 10844-10999 | [pfam01390](http://www.ncbi.nlm.nih.gov/entrez/query.fcgi?cmd=Search&doptcmdl=GenPept&db=cdd&term=pfam01390) | 107 | 75.88 | 2.37E-18 | 4 | 114 |
| R61 | 11000-11154 | [pfam01390](http://www.ncbi.nlm.nih.gov/entrez/query.fcgi?cmd=Search&doptcmdl=GenPept&db=cdd&term=pfam01390) | 107 | 69.72 | 4.64E-16 | 19 | 114 |
| C-term | 11439-11722 | [pfam01390](http://www.ncbi.nlm.nih.gov/entrez/query.fcgi?cmd=Search&doptcmdl=GenPept&db=cdd&term=pfam01390) | 107 | 63.55 | 3.99E-13 | 100 | 203 |

*MUC16 sequence published in Reference 21 was used for this BLAST analysis. Repeats highlighted in yellow were likely misnumbered in Reference 21. Repeats 62 and 63 were incomplete in Reference 21 and were not included in our analysis.

Screen shot from a BLAST search conducted on Repeat #11 is shown below followed by a zoomed-in image of the sequence analysis showing the four amino acids (grey letters) that are found in MUC16 tandem repeat but not the SEA sequence.


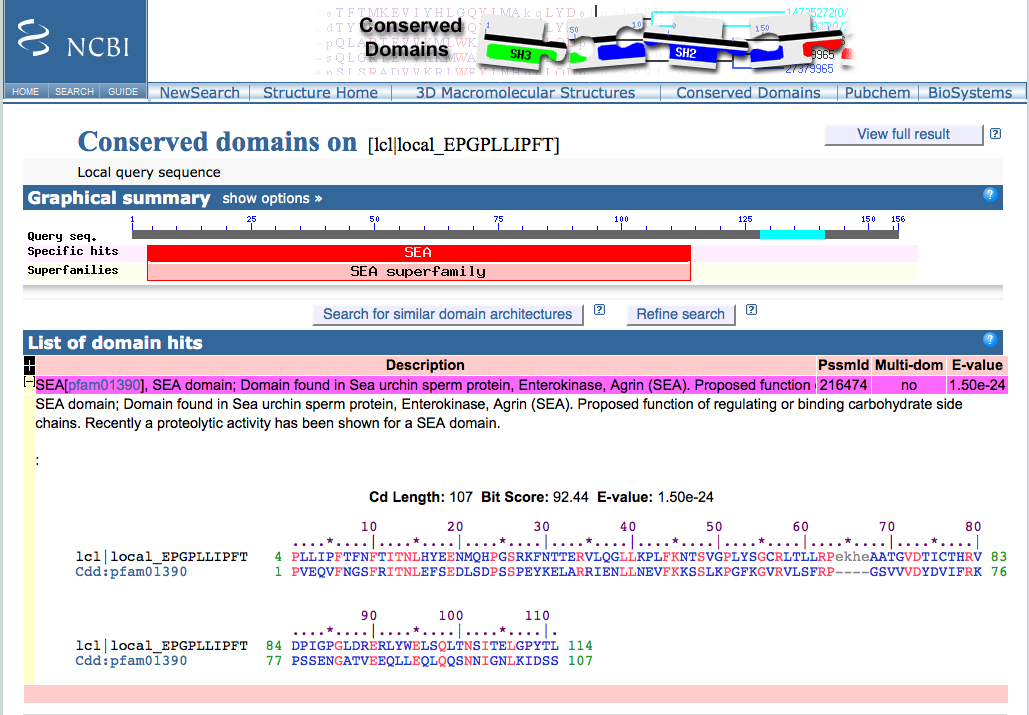


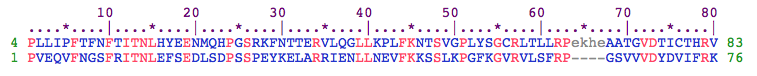


**SEA**

**MUC16 repeat**
